# Movable Kidney

**Published:** 1905-01-14

**Authors:** 


### MOVABLE KIDNEY.

We are very pleased to see in the current number of the *Practitioner* an emphatic protest from Sir Frederick Treves against the indiscriminate performance of nephropexy for movable kidney. The time is opportune for such an authoritative warning. Few if any of the operations of surgery have brought so much discredit to the craft as that of cutting down upon a movable kidney and attempting, by sutures or other means, to fix it in its proper position. Judged by its results nephropexy is one of the most unsatisfactory operations of surgery, and the sooner this is realised the better, for a large proportion of women (over a third of all adult women, according to Dr. Larrabee) have movable kidneys, and are consequently in perpetual danger if they complain of pain in the abdomen, as it is the fate of most women to do at some time in their lives, of being advised to undergo a serious and expensive operation, the results of which are very likely to be disappointing. One of the reasons given in defence of the operation is that the attendant risks are small. But, as Sir Frederick Treves says, an operation is neither justifiable nor commendable on the sole ground that it is accompanied by small risk. As he justly points out, the operation is far from being always successful; there is not one of the numerous methods proposed for performing it which can claim to be exempt from failure, and it is probable that a much smaller measure of success is obtained than is commonly admitted. In fact, the successes are given a prominence which is denied to the failures. We know of a case where a lady in good position suffered for two years after an operation for nephropexy from a sinus in the loin which would not heal, while the symptoms for which the operation had been performed were unrelieved—much to the discredit of the surgeon who operated and of surgery as a whole. Sir Frederick Treves would reserve the operation for cases in which there have been torsion symptoms, and in these instances he regards the operation as imperative. The symptoms of this complication occur in the form of sudden attacks of intense renal pain, vomiting, abdominal tenderness, and a varying degree of collapse. Some attacks are less abrupt, or may even be gradual in the onset and moderate in their manifestations. Some are relieved by posture. A temporary hydronephrosis may attend the attack or it may not. But the majority of cases of movable kidney, he thinks, can be most suitably and efficiently treated by means of a well-fitting and properly designed belt. With the one exception mentioned above, operation should be the last, and not the first, resource.

